# Unique Deep Radiomic Signature Shows NMN Treatment Reverses Morphology of Oocytes from Aged Mice

**DOI:** 10.3390/biomedicines10071544

**Published:** 2022-06-29

**Authors:** Abbas Habibalahi, Jared M. Campbell, Michael J. Bertoldo, Saabah B. Mahbub, Dale M. Goss, William L. Ledger, Robert B. Gilchrist, Lindsay E. Wu, Ewa M. Goldys

**Affiliations:** 1 ARC Centre of Excellence Centre for Nanoscale Biophotonics, University of New South Wales Sydney, Sydney, NSW 2052, Australia; j.campbell@unsw.edu.au (J.M.C.); s.mahbub@unsw.edu.au (S.B.M.); e.goldys@unsw.edu.au (E.M.G.); 2 Fertility & Research Centre, School of Clinical Medicine, University of New South Wales Sydney, Sydney, NSW 2052, Australia; michael.bertoldo@unsw.edu.au (M.J.B.); w.ledger@unsw.edu.au (W.L.L.); r.gilchrist@unsw.edu.au (R.B.G.); 3 School of Medical Sciences, University of New South Wales Sydney, Sydney, NSW 2052, Australia; dmgoss05@gmail.com (D.M.G.); lindsay.wu@unsw.edu.au (L.E.W.)

**Keywords:** oocyte, aging, morphology, machine learning, NMN

## Abstract

The purpose of this study is to develop a deep radiomic signature based on an artificial intelligence (AI) model. This radiomic signature identifies oocyte morphological changes corresponding to reproductive aging in bright field images captured by optical light microscopy. Oocytes were collected from three mice groups: young (4- to 5-week-old) C57BL/6J female mice, aged (12-month-old) mice, and aged mice treated with the NAD+ precursor nicotinamide mononucleotide (NMN), a treatment recently shown to rejuvenate aspects of fertility in aged mice. We applied deep learning, swarm intelligence, and discriminative analysis to images of mouse oocytes taken by bright field microscopy to identify a highly informative deep radiomic signature (DRS) of oocyte morphology. Predictive DRS accuracy was determined by evaluating sensitivity, specificity, and cross-validation, and was visualized using scatter plots of the data associated with three groups: Young, old and Old + NMN. DRS could successfully distinguish morphological changes in oocytes associated with maternal age with 92% accuracy (AUC~1), reflecting this decline in oocyte quality. We then employed the DRS to evaluate the impact of the treatment of reproductively aged mice with NMN. The DRS signature classified 60% of oocytes from NMN-treated aged mice as having a ‘young’ morphology. In conclusion, the DRS signature developed in this study was successfully able to detect aging-related oocyte morphological changes. The significance of our approach is that DRS applied to bright field oocyte images will allow us to distinguish and select oocytes originally affected by reproductive aging and whose quality has been successfully restored by the NMN therapy.

## 1. Introduction

In humans, the non-renewable reserve of oocytes is laid down during in utero development, where they must be maintained prior to ovulation and be ready for fertilization decades later. Given the decades that oocytes must persist in the ovarian environment, oocytes are highly vulnerable to aging, with impacts that occur well before other tissues [1,2]. Oocyte aging represents a key constraint on natural and assisted reproduction, yet the ability to objectively measure the impacts of aging on oocytes in a non-invasive manner is lacking. Currently, oocyte morphological analysis is undertaken using light microscopy by trained embryologists to provide a rough assessment of oocyte quality. However, visual assessment is highly subjective and poorly predictive [3]. Little work has been done on the impact of biological aging on oocyte morphology, with one study observing no difference for 20, 25, and 30 weeks of age in mice on zona pellucida thickness or periventricular space in mice [4]. An accurate methodology for assessing age-related differences in oocyte morphology is needed. This would have potential clinical utility (for optimizing oocyte selection) and enable new avenues of research for reversing age-related declines in oocyte quality.

Recently, artificial intelligence (AI) has been widely applied to objective biomedical image assessment for disease diagnosis and monitoring to enable the precise customization of treatment plans [5,6,7,8,9]. Deep learning strategies (machine learning algorithms that use multiple layers to progressively extract higher-level features from data) have been used to interpret electroencephalogram (EEG), electrocardiogram (ECG), magnetoencephalography (MEG), and magnetic resonance imaging (MRI) data, to improve reliability and precision [10]. This recent surge in interest has led to several attempts to apply AI methodologies to the assessment of embryo viability for human-assisted reproduction, although success has been variable [11,12,13]. AI methods have been proposed to automate sperm, and embryo assessment through morphology analysis such as time-lapse imaging [14,15,16,17,18].

In this study, we hypothesized that aging impacts oocyte morphological properties and generates a specific morphology signature in oocytes. As such, we applied a deep radiomic (i.e., relating to the algorithmic extraction of a large number of features from medical images) signature (DRS) method to detect age-related changes in oocyte morphology. DRS is an AI quantitative approach for automating the extraction of information from images to standardize the interpretation of medical imaging. Brightfield images of oocytes were segmented, and morphological features were extracted using deep structured nets which capture features including shapes and textures. This approach identifies image differences resulting from characteristics perceptible to the human eye, such as oocyte size and circularity. In addition, this technique can capture characteristics that are imperceptible by traditional visual inspection of oocytes, including the specific spatial distribution of image pixel intensities and pixel interrelationships, where there are no existing mathematical definitions with which they can be captured [19].

To discover the morphological DRS for the impact of maternal age on oocyte morphology, we used a novel combination of artificial intelligence methods, including deep learning [20,21,22], swarm intelligence [7,23,24], and discriminative analysis [25,26]. Subsequent to the DRS discovery, we applied our new DRS to brightfield microscopy images of oocytes obtained from aged mice that had been treated with nicotinamide mononucleotide (NMN). NMN is an orally deliverable metabolic precursor to the metabolic redox cofactor nicotinamide adenine dinucleotide (NAD+) and is essential for energy metabolism (via its involvement in the electron transport chain), DNA repair, and epigenetic homeostasis. We have previously shown that NMN positively impacts female reproductive aging [27]. In this way, we tested our hypothesis that the DRS would be sensitive to reversals of the impact of aging by comparing the morphology of old, NMN oocytes to that of young and old oocytes. To the best of our knowledge, this is the first study where age-related oocyte morphological changes have been quantified using artificial intelligence.

## 2. Materials and Methods

### 2.1. Oocyte Collection and NMN Treatment

The oocyte images used in this study were brightfield microscopy images that were collected during a recent study [27] but whose morphology was not previously analyzed. To recover metaphase II (MII) oocytes, aged (12-month-old) and young (4- to 5-week-old) C57BL/6J female mice were maintained in individually ventilated cages at 22 °C at 80% humidity at a density of 5 per cage, with ad libitum access to food and water. All water in this animal house was acidified to pH 3 with HCl to decrease microbial growth. The UNSW animal house maintained a 12 h light/dark cycle with lights on at 0700 and off at 1900. Experiments were carried out with prior approval of the UNSW Animal Care and Ethics Committee (ACEC) under ACEC number 18/133A. UNSW ACEC operates under strict animal ethics guidelines from the National Health and Medical Research Council (NHMRC) of Australia.

Aged females were treated with NMN in drinking water (2 g/L) for 4 weeks. Oocytes from young mice are of very high quality and have high developmental competence, as such no additional benefit is seen for NMN treatment and hence this was not examined. After 4 weeks, both aged and young females were treated with an intraperitoneal (i.p.) injection of pregnant mare serum gonadotrophin (PMSG; Folligon, Intervet, Boxmeer, The Netherlands) to stimulate follicle growth, followed by an i.p. injection of human chorionic gonadotrophin (hCG; Chorulon, MSD Animal Health, Sydney, Australia) 46 h later to induce ovulation. Young females were administered 5 IU PMSG and 10 IU hCG, whereas aged animals were treated with 10 IU PMSG and 10 IU hCG COCs were collected from oviductal ampullae using a 27-gauge needle and collected in HEPES-buffered α-minimum essential medium (α-MEM; GIBCO Life Technologies, Grand Island, NE, USA) supplemented with 3 mg/mL bovine serum albumin (BSA; Sigma Aldrich. St. Louis, MO, USA) 14–16 h after hCG injection. Oocytes were stripped of their cumulus cells with hyaluronidase (concentration, supplier, etc.). Non-degenerate, nominally healthy oocytes were then placed into equilibrated Hank’s balanced salt solution (HBSS) under paraffin oil for imaging. Finally, the number of collected oocytes from young, old, and old NMN-treated mice are 26, 21, and 29, respectively.

### 2.2. Microscopy Imaging

Standard brightfield microscopy imaging was performed on an Olympus iX83 system with a 40× oil objective (NA 1.15) and a Prime95B™ sCMOS (Photometrics) camera operated below −30 °C to reduce noise. The image size was 1200 × 1200 pixels.

## 3. Data Analysis

**Figure 1:**
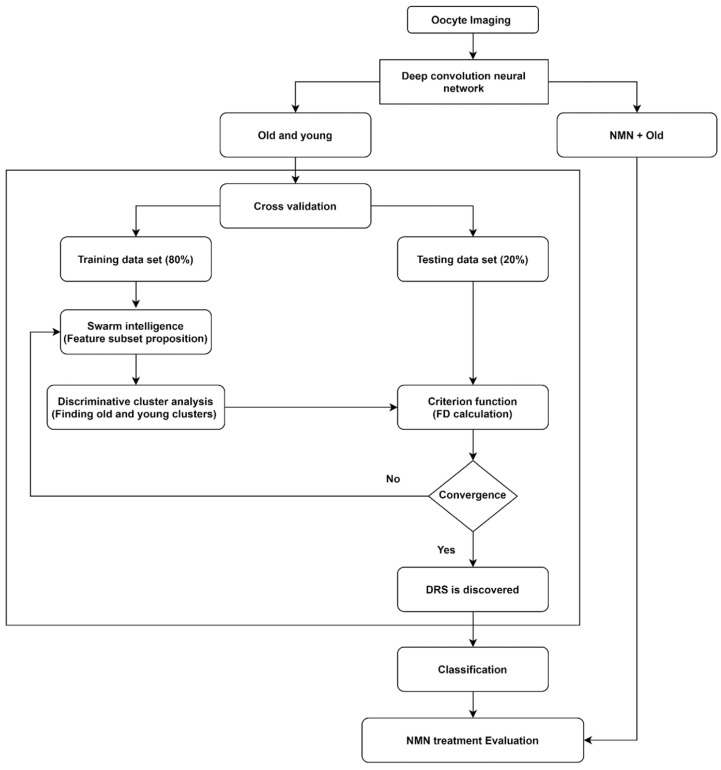
Data analysis methodology employed in this study.

We applied a novel artificial intelligence strategy combining deep learning, swarm intelligence, and discriminative analysis to images of mouse oocytes taken by bright field microscopy to create a highly informative deep radiomic signature (DRS) of oocyte morphology. The process of data analysis is illustrated in Figure 1. After bright field imaging, oocytes were segmented to isolate the image sections containing oocytes only. Then, oocyte images were augmented [28] to artificially expand the dataset by adding images that are intuitively equivalent to the original images (details in Section 3.1. Data augmentation). Next, old and young oocytes were provided to the deep learning nets which were constructed to extract the deep information where several (here N = 3) bespoke deep learning nets with different structures and resolutions were implemented to extract accurate, data-driven image feature information (details in Section 3.2. Deep convolutional neural network). Further, the information associated with old and young oocytes was used to discover the DRS.

To discover DRS, we used discriminative analysis based on the feature subset iteratively selected from the pool of features by swarm intelligence, a technique that draws on the collective behavior of a group of naïve agents [29] (details in Section 3.3. Swarm intelligence). To this end, data points were partitioned into training data set (80% of data) and testing data set (20% of data) through a cross-validation process. The DRS can take advantage of numerous image characteristics including size, circularity, the spatial distribution of variations in pixel intensities, and pixel interrelationships. Using the training data set, feature values from the oocyte groups under consideration (here, young and old oocytes) are represented in a 2-D discriminative space spanned by two canonical variables providing the highest separation of these clusters measured by the Fisher distance (FD) (ratio of between-cluster and within-cluster variances) [30]. The canonical variables in our work are the optimal linear combinations of the utilized features. Further, the testing data points are projected onto this 2D discriminative space, and their Fisher distance (FD) is evaluated. This is followed by the next round of feature selection (new subset) by swarm intelligence and discriminative cluster analysis where the maximization of FD calculated on the testing data serves as the criterion in the swarm intelligence process. This iterative search for improved feature subsets is carried out until the algorithm achieves satisfactory convergence of FD with respect to its changes between consecutive swarm intelligence iterations. This DRS was then used to obtain the support vector machine classifier (details in Section 3.4) allowing us to distinguish old vs young oocytes which were rigorously cross-validated and finally we used that classifier to evaluate the NMN + old oocytes and produced the assessment of the NMN treatment outcomes.

### 3.1. Data Augmentation

Image augmentation [28] is a technique to artificially expand the dataset by adding images that are intuitively equivalent to the original images, in this case, images of oocytes that have been rotated by various angles, or reflected along various axes—as the oocyte orientation on the microscope is irrelevant This leads to enhancements in both the quantity and diversity of the data for training models, improving the performance and ability of the model to generalize. With image augmentation, CNN is able to learn features that are invariant with respect to their location in the image and image orientation. Image augmentation can aid the model in learning features that are invariant to intuitively acceptable image transforms such as left-to-right orientation to top-to-bottom ordering, etc.

In this work, we applied image augmentation to deal with the limited number of training images and enhance the performance of the CNN for discovering the DRS. For data augmentation, oocyte images and their mirror reflections were rotated at different angles (45°, 90°, 13°, 18°).

### 3.2. Deep Convolutional Neural Network

A convolutional neural network (CNN) is a deep learning strategy that automatically performs feature identification [31]. To identify features, the CNN carries out several procedures called convolutional layers that are sequentially applied to an input image to learn the features that in traditional algorithms were derived based on mathematical feature definitions [32]. This independence from mathematical definitions that represent prior knowledge in the feature extraction is the major advantage of CNN. A convolutional layer contains a number of sub-procedures called filters where image convolution with specific filter arrays is carried out to extract image information. As an example, a filter with a 3 × 3 array whose parameters are all 1/9 computes the average of 9 pixels of the image after convolution. The number of filters can vary from layer to layer. The parameters of these arrays could be taken from the literature, where one can source filter arrays from convolutional nets previously trained on large image data sets [33]. This is possible because some image features, such as edges, shapes, corners, and intensity are common in a wide range of images, enabling knowledge transfer [33]. Such filter sharing adds efficiency while maintaining good generalization [34]. Alternatively, the filters could be learned from the dataset through a learning process [35]. Training is the step where the network learns from the data. Each filter array is assigned with random parameters and the classifier goes a forward pass based on the data to predict the class labels. Further, the predicted class labels are compared against the actual class labels and an error is calculated. This error is subsequently back propagated across the network and parameters are revised accordingly [35].

To apply the filter to the input image, the filter array is moved across the width and height of the input image, and the dot products between the input image and filter array are computed at every spatial position. The output of the filter is another image of reduced size compared with the input image to the filter. Each convolutional layer is immediately followed by two other procedures called activation and pooling layers. The specific activation layer used in this work referred to as a “rectified linear unit (ReLU)” removes negative pixels in the input image replacing them with zeros but retains all positive pixels. The role of the pooling layer is to reduce the spatial size of the input image by a chosen pooling operation. Here, we used max-pooling where each 3 × 3 image tile is replaced by the maximum value in that tile. The activation and pooling layers lead to more effective training, by eliminating negative values, down-sampling (making images smaller) and reducing the number of parameters that the network needs to learn.

The output of each convolutional layer is a modified image used as the input to the next convolutional layer. The convolution, ReLU, and pooling processes are repeated until the final high-level information about the image (image features obtained through deep learning) is extracted at different resolutions depending on the filters and the specifics of convolutional layers employed in the nets which alter the data with the intent of learning the features [34]. After learning the features by using several convolutional layers, the CNN typically shifts to classification through the next set of protocols called “fully connected layers” as in a standard multilayer neural network approach [35] however this leads to many unknown parameters that can only be defined through training on thousands of images [36], which is extremely challenging for clinical experiments and their limited number of images. Deep networks that only have a limited amount of training data suffer from a reduction in accuracy and generality power of the model, especially when the depth (number of layers) of the network increases [37].

In this study, three CNNs (Net1, Net2, and Net3) were used to extract image features. Net 1 consisted of 152 convolutional with specific filters taken from ResNet [38]. The parameters in the first 151 convolutional layers were taken exactly as in ResNet and then Net 1 was trained using oocyte data to correct the deep features based on the actual oocyte features. Net 2 contained 22 convolutional layers. It was built by drawing on the GoogLe net [39] where 21 layers used the filters from GoogLe and the 22-nd layer was fine-tuned by the training data set of the oocytes. Such fine-tuning corrects the net parameters to align them more closely with oocyte morphological features. Net 3 was developed specifically for this study. It had 5 convolutional layers, where the first 4 convolutional layers used the filters from the Krizhevsky net [40]. The fifth convolutional layer had 96 filters with a size of 3 × 3 pixels; these filters were trained using the oocyte data set. In the end, by applying these nets, we were able to generate N_f_~6000 of deep learning image features for our dataset. We did not use CNNs for feature classification, this was carried out by the method of swarm intelligence detailed below.

### 3.3. Swarm Intelligence

Swarm intelligence is a methodology inspired by the evolution of a group of simple information-processing interacting agents [23]. In this approach, the naive artificial agents (in this case feature subsets) are iteratively evolved according to a pre-set evolution rule attempting to find the highest FD as a criterion optimization problem [41]. First, a population of the agents (feature subsets) is generated, and the FD is assessed for each of these agents, and then the agents are repeatedly updated according to a defined evolutionary strategy until the convergence condition for FD is satisfied.

In this study, we chose the agents to be candidate feature subsets of all available features generated by our CNN (N_f_~6000), and 50 agents were used. We have run the swarm intelligence process multiple (19) times to optimize the number of features K underpinning the deep radiomics signature, starting from K = 2 to K = 20.

### 3.4. Support Vector Machine Classifier

In this work, we used a support vector machine classifier (SVM), a strong supervised method that can deal with sparse data with a limited risk of being overfitted. In this approach, a hyperplane is formed [26,42,43] with maximum margins in the high dimensional spectral feature space to separate data points into the classes under consideration (here, young and old oocytes). This classifier defines the data label based on a linear predictor function [44]. Then the classifier is trained using optimal DRS to predict the pre-defined data labels (here, old and young). To train our SVM classifier the method of 10-fold cross-validation was employed, and the classifier performance was evaluated using nested cross-validation and bootstrapping [45].

## 4. Results

**Figure 2:**
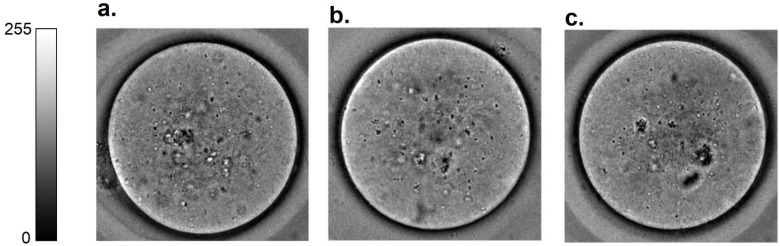
Representative brightfield images of oocytes from (**a**) young (4–5 weeks), (**b**) aged (12 months), and (**c**) aged animals treated with NMN. Oocytes from the different groups were morphologically indistinguishable by visual inspection.

Our approach obtained comprehensive morphological information from brightfield images of oocytes obtained following the super-ovulation of young (4–5 weeks) and reproductively aged (12 months) mice. Following the training of our DRS on these images of oocytes from young and aged animals, we applied this system to images of oocytes from a separate group of reproductively aged (12 months) mice that were treated with NMN (See Methods Section 2.1). Figure 2 shows representative brightfield oocyte images of oocytes from these three groups of animals. All oocytes were non-degenerate and therefore nominally healthy in terms of reproductive competence, and were not morphologically distinct by visual inspection.

**Figure 3:**
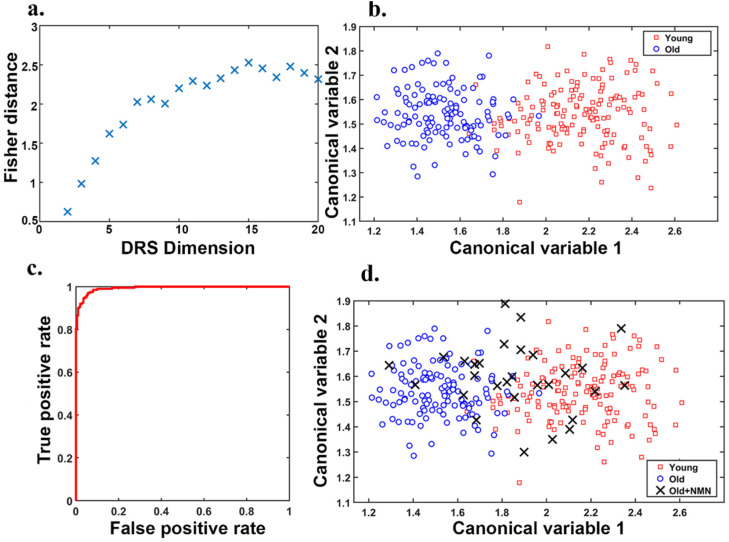
(**a**) Improvement in DRS discriminative power measured by Fisher Distance (FD) with an increasing number of DRS features. (**b**) Discrimination of old and young oocyte morphology using our optimal DRS (points represent oocytes with data augmentation: Number of old data points after augmentation = 126, Number of young data points after augmentation = 156), Figure with no data augmentation is shown in Supplementary Figure S4 (**c**) Classification performance curve of receiver operating characteristics (ROC) for our optimal DRS with 15 features. (**d**) DRS discrimination of oocytes from young and old animals as shown in (**b**) overlaid with oocytes from aged animals treated with the NAD precursor NMN (Number of NMN data points = 29), each data point represents an individual oocyte.

After applying image augmentation and a deep convolutional learning approach, the DRS was discovered using a combination of the swarm intelligence method with discriminative analysis which was cross-validated using a testing dataset (Supplementary Figure S1, further information in Supplementary Material Section S1). The number of features in the swarm intelligence subsets (DRS dimension, Figure 3a) was independently varied and optimized, and DRS was constructed using 15 features (30% from Krizhevsky net, 10% from Google net, and 60% from Resnet). As shown in Figure 3b, this DRS allowed us to clearly separate clusters of young (red data points) and old oocytes (blue data points), highlighting a significant difference in morphology that could be automated for standardization of assessment. The final DRS based on 15 features was used to train our support vector machine classifier (further details supplementary material Section S3). To this aim, 10-fold cross-validation was employed, and the classifier performance was evaluated using nested cross-validation and bootstrapping (further information in Supplementary Material Section S2, Supplementary Figures S2 and S3). The corresponding receiver operating characteristic (ROC) graph determining the DRC classification performance (Figure 3c) showed it has an accuracy of 92.2 ± 3.3 and an area under the curve (AUC) of 0.99.

Brightfield images of oocytes from older, NMN-treated mice from our recent work [27] were taken concurrently with oocytes used to define the DRS (Figure 1) these oocytes were not used for the development of the canonical variables applied, and are fully independent. We showed that data points representing oocytes from NMN-treated aged mice (black crosses) moved away from the cluster of oocytes from untreated age-matched controls (blue circles) toward the young cluster (red squares; Figure 3d). Overall, ~55% of NMN-treated oocytes had morphological properties that exactly correlated to the young cluster, ~25% were very close to the young cluster and 20% retained the morphological properties of oocytes from old mice. Morphological features of oocytes from NMN-treated animals were subsequently fed to the support vector machine classifier trained with our DRS. This resulted in 60% of oocytes being sorted into the young group and 40% into the aged group. As NMN is known to restore ‘youthful’ characteristics and health to oocytes [27], this shows that the DRS is sensitive to changes induced in the biological age of oocytes induced by geroprotective interventions. Furthermore, as detailed in [27,43], hyperspectral imaging enabled the quantification of specific autofluorophores, including the key metabolic cofactor NAD(P)H. Remapping these findings to the results of the morphology analysis showed that the NAD(P)H levels of Old + NMN oocytes classified as young had the same NAD(P)H profile as actual young oocytes, while those classified as old matched other old oocytes (Supplementary Figure S5). Additionally, NAD(P)H was significantly elevated in young, sorted oocytes compared to old sorted oocytes. This provides direct biochemical evidence that the DRS is genuinely sensitive to oocyte age and quality.”

## 5. Discussion

Morphology plays a significant role in developmental biology and is strongly affected by the cell’s microenvironment and response to biophysical and environmental factors [46]. Reliable oocyte morphology quantification indicative of oocyte biological (as opposed to chronological) age would be of significant utility to advise clinical decision-making. As well as facilitating gamete selection, it would enable women undergoing cryopreservation of oocytes for fertility preservation to receive an informed estimate of when sufficient oocytes have been collected to reasonably guarantee future success mitigating the likelihood of both collecting too few oocytes and subjecting women to additional, unnecessary rounds of stimulation and collection. However, there are no options with sufficient consistency and precision for widespread uptake for oocyte morphology analysis. Conventional morphological analysis for oocyte competency scoring is mostly limited to visually visible features, including that oocytes should have a clear, moderately granular cytoplasm, without inclusions, a small perivitelline space with a single unfragmented first polar body, and a round clear zona pellucida [3]. Generally, findings have been mixed with regards to whether conventional scoring of oocyte morphology can be prognostic for oocyte quality assessment in terms of fertilization and pregnancy [47]. A systematic review of fifty studies that investigated the impact of single or multiple oocyte features on in vitro fertilization (IVF) outcomes did not find any visible features with unanimous prognostic value for the further developmental competence of oocytes. More promising results were found for complex classification systems which considered multiple features. Extreme variability has also been observed between individual assessors and facilities applying scoring systems [48,49]. Overall, while conventional assessment of oocyte morphology has demonstrated that morphology has the potential to indicate the reproductive potential and quality of an oocyte [50], this promise has been difficult to realize through manual observations.

This study introduced a standard quantitative approach to assess oocyte morphology properties named DRS which enabled oocytes to be sensitively categorized according to their age category. We used brightfield images of oocytes from young and reproductively aged animals to extract morphological features and develop a DRS for the morphology of aging in oocytes. In this study, we demonstrated that a DRS can differentiate young and old oocytes with 92.2 ± 3.3 accuracy. To extract features and create a comprehensive feature bank, three different nets with different structures were employed. We used extremely deep (Res net), deep (Google net), and moderately deep (Krizhevsky net) structures to capture features with different resolutions. DRS discovered using swarm intelligence shows that features have been selected from all three nets. This demonstrates that the efficacy of using three networks while using only one net may result in suboptimal DRS and consequently lower classification performance. Segmentation of oocyte images from the background was performed manually without subjective selection and all available oocyte images were used for subsequent analysis. Oocyte segmentation can potentially be performed automatically, which would classify the DRS methodology as fully automated.

Further, we analyzed the effect of NMN on oocyte morphology when it is used to treat aged animals. NMN treatment supports the generation of the redox cofactor nicotinamide adenine dinucleotide, which is essential to fundamental metabolic processes including glycolysis and the TCA cycle, and also acts as a substrate for proteins involved in DNA repair and epigenetic maintenance, such as members of the poly-ADP-ribose (PARP) and sirtuin family. We recently showed that NAD+ levels decline with age, impairing oocyte function, and that restoration of NAD+ levels through treatment with its metabolic precursor NMN could restore oocyte quality and functional fertility in aged animals [27]. As the availability of competent oocytes is a rate-limiting factor in human reproduction their rejuvenation, and subsequent identification of high-quality oocytes is an important goal for reproductive healthcare in the context of an aging population. In this study, we showed that in NMN-treated aged animals, oocyte morphological properties were restored to those of young oocytes in 60% of cases. Those oocytes from old animals with young morphological properties also had the same levels of the key metabolite NAD(P)H as oocytes from young animals, while conversely, oocytes with old morphological properties had low levels of NAD(P)H corresponding to the levels seen in oocytes from old untreated animals (Supplementary Figure S5). This provides biochemical confirmation that the DRS is genuinely sensitive to changes in oocyte quality. As well as adding to the growing evidence that NMN may address reproductive aging in females [27], these findings have implications for research into oocyte quality and aging.

Our results indicate that a combination of modern artificial intelligence methods of deep learning and swarm intelligence coupled with discriminative analysis produces a DRS capable of recognizing age-related changes in oocyte morphology. Our methodology outperforms the conventional oocyte morphology analysis as it is automatic, objective, fast and can extract and consider specific features which are undetectable to human vision and there is no particular mathematical definition for them. As well as the potential for optimizing oocyte selection in clinical practice, DRS could greatly accelerate the efficiency of research in oocyte quality and aging. As the only reliable measures of oocyte quality are future outcomes (e.g., fertilization, blastocyst development, implantation, and/or pregnancy), which are time- and labor-intensive, experimentation on interventions to optimize oocyte quality is severely curtailed. The direct measurement of apparent oocyte age given by our DRS would enable high throughput investigations of potential interventions to restore oocyte competence in the face of reproductive aging. This study was performed by bright-field microscopy which is the plainest of all the optical microscopy illumination methods and involves only basic equipment. Bright-field imaging is routinely used in reproduction laboratories and therefore our methodology has high translatability.

This study was conducted as a proof of principle using images from mouse oocytes, as such a limitation is that it will be necessary to evaluate this technology in images of human oocytes to test its relevance for clinical practice. Although DRS is shown to be statistically robust with respect to the number of oocytes allowing the extraction of strong morphological signatures (backed up with statistical evidence in the manuscript), enhancing the number of studied subjects in a data set could improve the strength of the discovered DRS. Additionally, the images used were 2-dimensional snapshots, and the orientation of oocytes will have resulted in some potentially informative features being present or absent in different images, reducing their utility. It is possible the model could be improved through the application of “z-stack” reconstructions. Furthermore, this study showed the possibility of extracting a unique morphological signature of age and was calibrated with known young and aged oocytes. Future application of the DRS as a tool for assessing the competency of aged oocytes for successful pregnancy will require us to follow up with experiments that directly demonstrate its association with this primary outcome.

The success rate of reproduction depends highly on oocyte quality and the current pregnancy rate per retrieved oocyte is estimated at 4.5%. Therefore DRS, as a potential non-invasive approach to predict human oocyte developmental potential, has promise in reliably improving the prediction of viability and blastocyst formation [51] for future clinical practice.

## Data Availability

Data are available upon request.

## Supplementary Materials

The following supporting information can be downloaded at: https://www.mdpi.com/article/10.3390/biomedicines10071544/s1. Ref. [52] is citied in supplementary materials.
