# Supplementary material for: Unique Deep Radiomic Signature Shows NMN Treatment Reverses Morphology of Oocytes from Aged Mice
Source: Biomedicines. 2022 Jun 29;10(7):1544. doi: 10.3390/biomedicines10071544 (PMC9313081; doi:10.3390/biomedicines10071544)
Supplement: Supplementary file 1 [file biomedicines-10-01544-s001.zip › biomedicines-1714442-SM.pdf]

Supplementary materials

NMN treatment reverses unique deep radiomic signature morphology of oocytes from aged mice

Supplementary material section S1:

The robustness of DRS in separating young and old oocytes was evaluated based on cross validation through data partitioned into 80% a training set (80% of data) used to create the discriminative space and the remaining 20% data formed the testing dataset for discriminative space evaluation. As shown in Figure S1, DRS formed two clearly separate clusters of young (circle red data points) and old oocytes (circle blue data points) using training data points. Next, testing datapoints which had put aside was reflected to the same space. This shows that DRS could successfully identify young (crossed red data points) and old (crossed blue data points) testing data points as they were located correctly on the appropriate clusters, which highlights the strength of DRS to deal with unseen data.

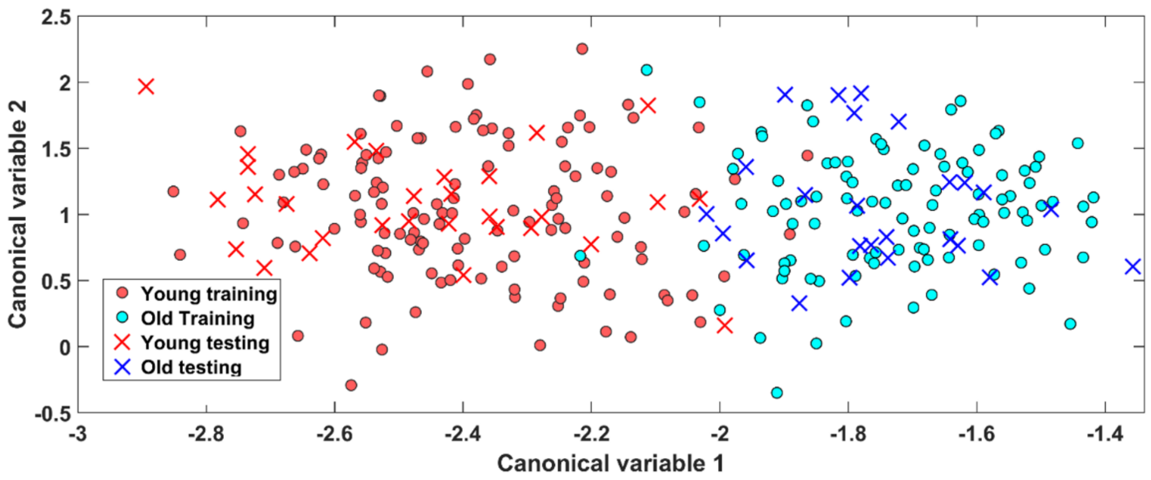

Supplementary Figure S1. DRS validation through testing and training cross-validation process

Supplementary material section S2:

To rigorously validate the classifier performance and minimize the risk of overfitting , we used nested cross validation as shown in Supplementary Figure S2, which provides unbiased performance assessment using external validation test[1]. To this end, data were split into 5 subsets (each 20% of data) [1]. One subset was put aside as a nest for external classifier validation at a time and SVM classifier was constructed based on the remaining. To train SVM classifier, internal 10-fold cross validation was employed. To this end, training data were partitioned into 10 randomly selected folds of approximately equal size. One fold was used to validate the SVM model trained based on the remaining subsets. This process was repeated 10 times[2]. Once the classifier was trained, nested subset was used to calculate accuracy. This is repeated five times to use all five subsets which led to five classifiers each of which with a specific accuracy. Averaging all 5 accuracies produced the nested accuracy of 92.2±3.3.

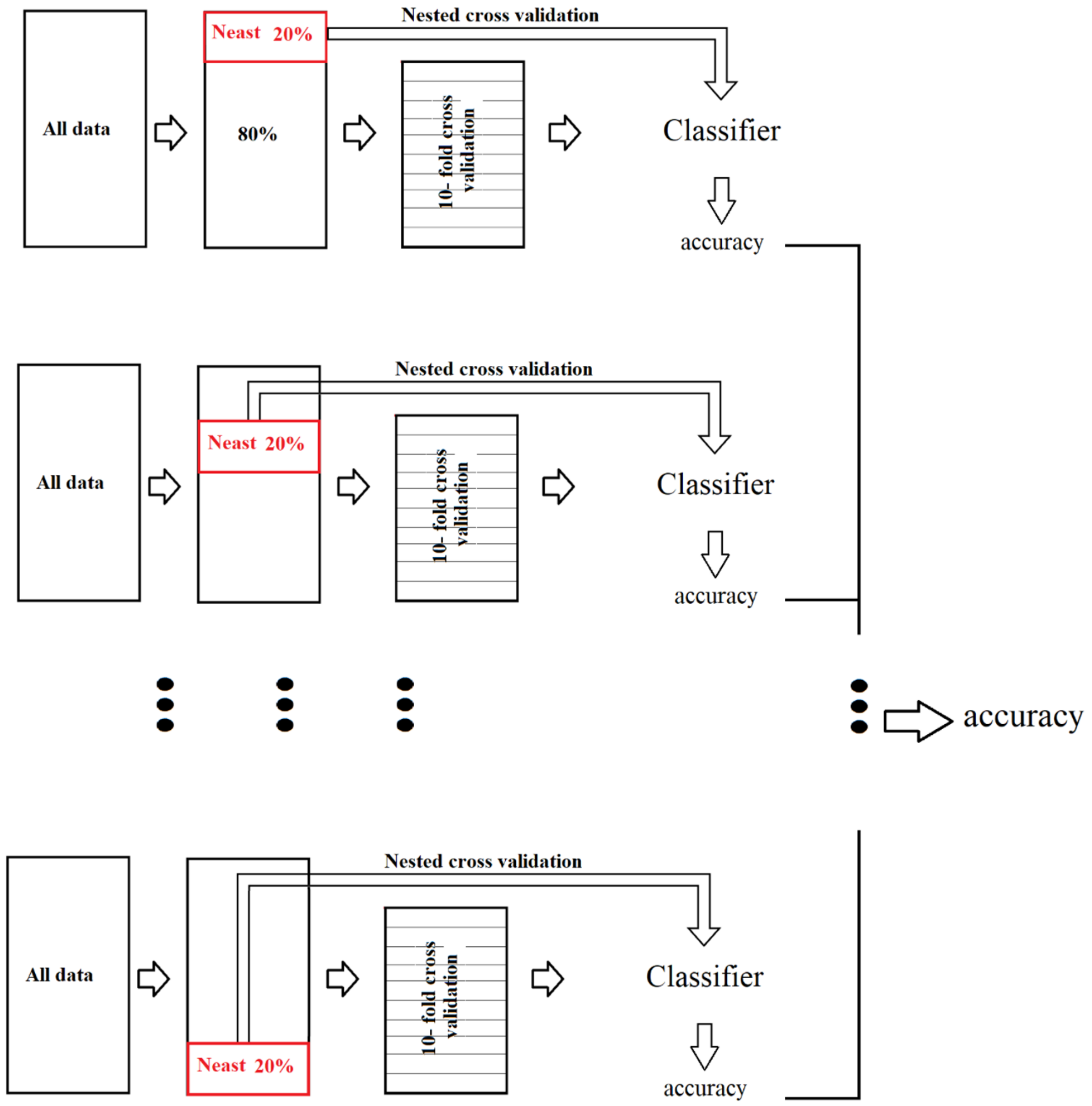

Supplementary Figure S2. Nested cross validation of our classifier

To estimate the sampling error due to relatively small number of oocyte images, the approach of bootstrapping was used[3]. The data points were randomly resampled 100 times with the replacement from our original set of observations and the corresponding ROC curves were obtained. Fig.S3 shows 95% confidence interval associated with ROC curve with AUC=0.99±0.01.

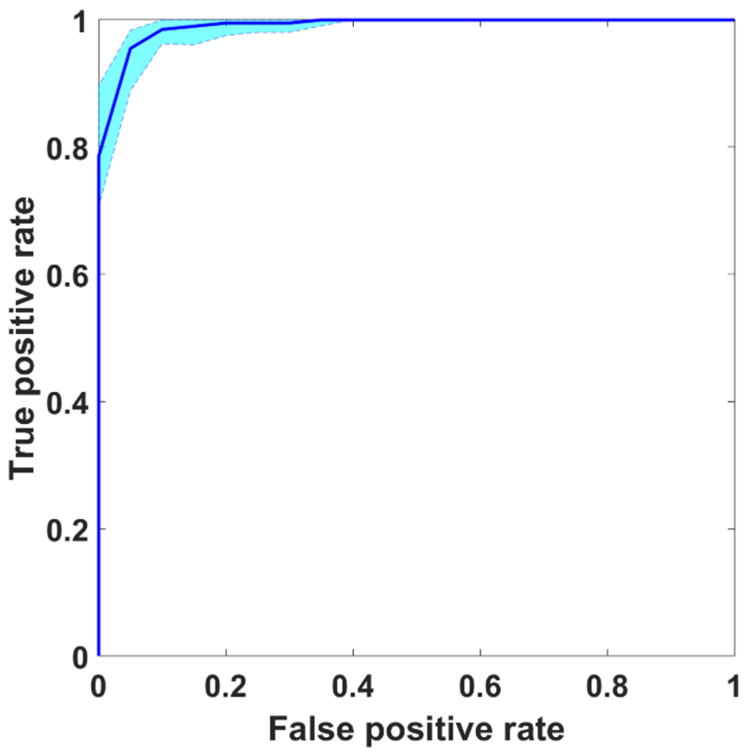

Supplementary Figure S3. ROC curve with the 95% confidence interval indicated.

Supplementary Figure S4 shows discrimination of old and young oocyte morphology using our optimal DRS with no data augmentation.

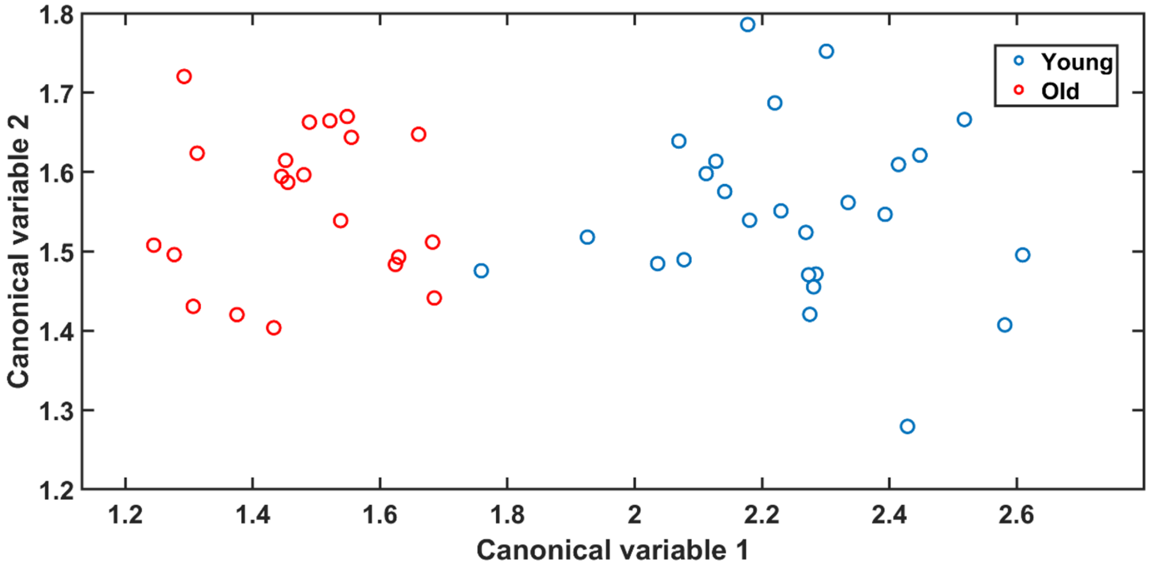

supplementary figure S4. discrimination of old and young oocyte morphology using our optimal DRS with no data augmentation.

Supplementary material section S3: Classifier selection

To select the best classification approach, different classifier algorithms were trained and compared based on area under ROC curve (AUC). SVM and quadratic classifiers showed higher performance with AUC~1 compared with Tree (AUC= 0.84), naïve base (AUC=0.96) and K nearest neighbor or KNN (AUC=0.91). Finally, SVM classifier was selected as it used linear kernel, which results in lower risk of be overfitting.

Supplementary material section S4: NAD(P)H abundance plot

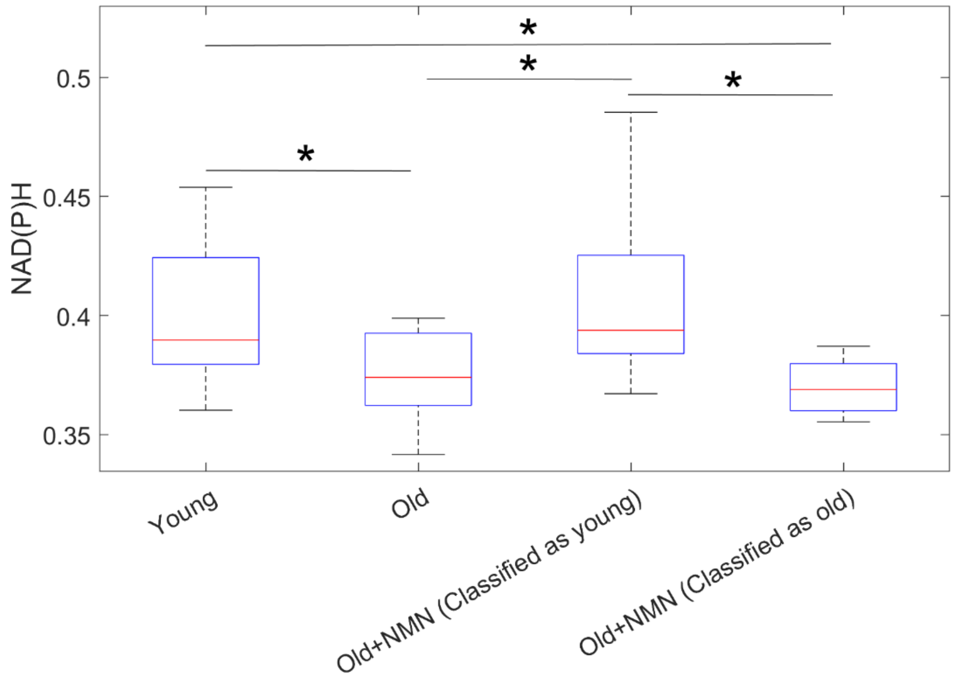

Supplementary figure S5. Oocyte NAD(P)H abundance from young, old, Old+NMN(classified as young) and Old+NMN(classified as old). (* represents p<0.05).

Supplementary References:

1. Vabalas, A., et al., *Machine learning algorithm validation with a limited sample size.* PloS one, 2019. **14**(11): p. 1-20.
2. Habibalahi, A., et al., *Novel automated non invasive detection of ocular surface squamous neoplasia using multispectral autofluorescence imaging.* The ocular surface, 2019. **17**(3): p. 540-550.
3. Efron, B. and R.J. Tibshirani, *An introduction to the bootstrap*. 1994: CRC press.
